# Persistent Coxiella burnetii Infection in Mice Overexpressing IL-10: An Efficient Model for Chronic Q Fever Pathogenesis

**DOI:** 10.1371/journal.ppat.0040023

**Published:** 2008-02-01

**Authors:** Soraya Meghari, Yassina Bechah, Christian Capo, Hubert Lepidi, Didier Raoult, Peter J Murray, Jean-Louis Mege

**Affiliations:** 1 Unité des Rickettsies, CNRS UMR 6020, Institut Fédératif de Recherche 48, Université de la Méditerranée, Faculté de Médecine, Marseille, France; 2 Department of Infectious Diseases, St. Jude Children's Research Hospital, Memphis, Tennessee, United States of America; Johns Hopkins School of Medicine, United States of America

## Abstract

Interleukin (IL)-10 increases host susceptibility to microorganisms and is involved in intracellular persistence of bacterial pathogens. IL-10 is associated with chronic Q fever, an infectious disease due to the intracellular bacterium Coxiella burnetii. Nevertheless, accurate animal models of chronic C. burnetii infection are lacking. Transgenic mice constitutively expressing IL-10 in macrophages were infected with C. burnetti by intraperitoneal and intratracheal routes and infection was analyzed through real-time PCR and antibody production. Transgenic mice exhibited sustained tissue infection and strong antibody response in contrast to wild-type mice; thus, bacterial persistence was IL-10-dependent as in chronic Q fever. The number of granulomas was low in spleen and liver of transgenic mice infected through the intraperitoneal route, as in patients with chronic Q fever. Macrophages from transgenic mice were unable to kill C. burnetii. C. burnetii–stimulated macrophages were characterized by non-microbicidal transcriptional program consisting of increased expression of arginase-1, mannose receptor, and Ym1/2, in contrast to wild-type macrophages in which expression of inducible NO synthase and inflammatory cytokines was increased. In vivo results emphasized macrophage data. In spleen and liver of transgenic mice infected with C. burnetii by the intraperitoneal route, the expression of arginase-1 was increased while microbicidal pathway consisting of IL-12p40, IL-23p19, and inducible NO synthase was depressed. The overexpression of IL-10 in macrophages prevents anti-infectious competence of host, including the ability to mount granulomatous response and microbicidal pathway in tissues. To our knowledge, this is the first efficient model for chronic Q fever pathogenesis.

## Author Summary

The interaction between immune system and invading bacteria is sufficient to eradicate microorganisms in the majority of bacterial infections, but the suppression of the microbicidal response leads to reactivation or chronic evolution of infections and to bacterial persistence. Coxiella burnetii, an obligate intracellular bacterium, is responsible for Q fever. This infectious disease is characterized by a primary infection that may become chronic as endocarditis in patients with valvular damage and immunocompromised patients. Clinical and in vitro studies have suggested a role for interleukin-10 in the chronic evolution of Q fever. However, an efficient mouse model for chronic Q fever pathogenesis, which could serve as a platform for anti–C. burnetii drug or immunotherapy development, is lacking. Here we use transgenic mice with constitutive overexpression of interleukin-10 in the macrophage lineage to study C. burnetii infection. We report an efficient mouse model for chronic Q fever pathogenesis, which associates high levels of specific antibodies, sustained tissue infection, and reduced granuloma formation, as in human Q fever. We also find an anti-inflammatory transcriptional program and altered expression of chemokines in infected tissues.

## Introduction

The interaction between innate/adaptive immune system and invading bacteria is sufficient to eradicate microorganisms in the majority of bacterial infections. This microbicidal response is based on inflammatory cytokines, such as interferon (IFN)-γ and tumor necrosis factor (TNF), which control the expression of cytokines and chemokines and the production of toxic metabolites [1]. The suppression of the microbicidal response due to genetic disorders leads to reactivation or chronic evolution of infections and to bacterial persistence [2]. In addition, immunosuppressive treatments and anti-inflammatory cytokines such as interleukin (IL)-10 or transforming growth factor (TGF)-β may also disarm microbicidal responses and contribute to chronic evolution of bacterial infectious diseases [1,3].

IL-10 is known to increase host susceptibility to numerous intracellular microorganisms and is involved in the persistence of bacteria such as Bartonella quintana or Mycobacterium tuberculosis [3,4]. *Coxiella burnetii* is an obligate intracellular bacterium that replicates in macrophages (Mφ) and is responsible for Q fever. The disease is characterized by a symptomatic primary infection in a minority of individuals, which may become chronic as culture-negative endocarditis in patients with valvular damage and immunocompromised patients [5]. The diagnosis of chronic Q fever is based on the presence of high titers of anti-C. burnetii antibodies, and bacteriological methods are of interest to study cardiac valve specimen [6]. In chronic Q fever, IL-10 is overproduced [7], and in patients with acute Q fever and valvulopathy, the risk to develop Q fever endocarditis is related to IL-10 overproduction [8]. IL-10 interferes with Mφ activation through the inhibition of transcription of inflammatory genes [9] and enables Mφ to support C. burnetii replication [10]. IL-10 also blocks maturation of C. burnetii–containing phagosomes in monocytes from patients with Q fever endocarditis [11].

While clinical and in vitro studies have suggested a role for IL-10 in the evolution of Q fever, an efficient mouse model for chronic Q fever pathogenesis, which could serve as a platform for anti-C. burnetii drug or immunotherapy development, is lacking. In transgenic mice that overproduce IL-10 in the T-cell compartment, BCG clearance is impaired [12], but this model is inappropriate for studies of Q fever pathogenesis because multiple phenotypes complicate the analysis of Mφ-bacterium interaction. Similarly, infection of IL-10-deficient mice is uninformative for studies of chronic infections because C. burnetti–infected humans do not lack IL-10. A more robust model is described here that applies transgenic mice with constitutive overexpression of IL-10 in Mφ lineage (macIL-10tg mice) [13]. We report an efficient mouse model for chronic Q fever pathogenesis, which associates high levels of specific antibodies, sustained tissue infection, and reduced granuloma formation, as in human Q fever. We also found an anti-inflammatory transcriptional program associating increased expression of arginase-1, decreased expression of IL-12p40 and IL-23p19, and altered expression of chemokines in infected tissues.

## Results

### Persistent C. burnetii Infection in macIL-10tg Mice

**Figure 1:**
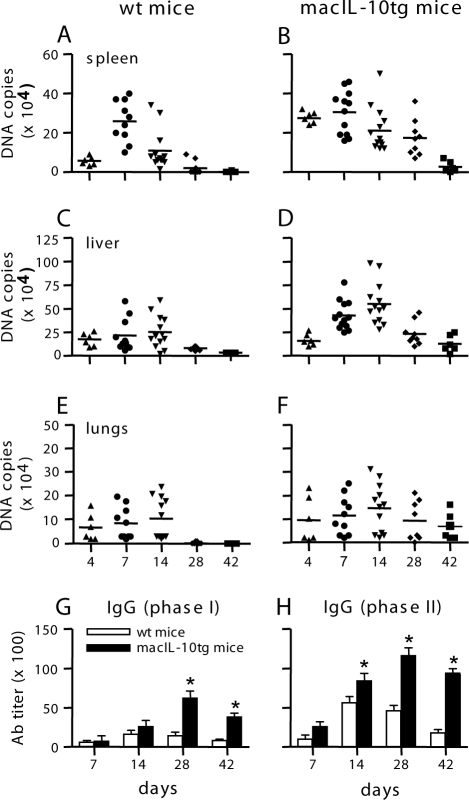
C. burnetii Infection in WT and macIL-10tg Mice (A–F) Tissue bacterial burden was measured by qPCR. The results are expressed as the number of C. burnetii DNA copies for each infected mouse. Bars represent the median of values for each time point. (G–H) The titer of specific Abs directed against C. burnetii in phase I and phase II was assessed by immunofluorescence using inactivated C. burnetii. The results are expressed as mean ± SD of at least five mice/time point. *p < 0.05.

When wild type (wt) and transgenic mice were injected with 5 × 10^5^ organisms by the intraperitoneal route, mortality or morbidity was not observed up to 60 d. The infection was assessed by qPCR in tissues and measurement of circulating specific antibodies (Abs) by immunofluorescence. Tissue infection was maximum at days 7 and 14 post-infection in wt and transgenic mice (Figure 1). At day 28 post-infection, only residual organ bacterial levels were observed in wt mice, whereas the infection of spleen, liver, and lungs was persistent in macIL-10tg mice, particularly for the lungs (*p* < 0.05). At day 42 post-infection, C. burnetii was completely cleared from the spleen, liver, and lungs of wt mice, but bacterial DNA was still present in the spleen, liver, and lungs from transgenic mice: the difference was significant (*p* < 0.05). After 60 d, no bacterial DNA copies were detected in spleen, liver, and lungs from wt and transgenic mice (unpublished data). The infection of mice was also studied through specific humoral response. In wt mice, the titer of IgG specific for phase I *C. burnetii* (Figure 1G) and phase II C. burnetii (Figure 1H) increased transiently. In macIL-10tg mice, the titer of specific IgG for phase I and phase II C. burnetii (Figure 1G and 1H, respectively) was high as compared with those found in wt mice (*p* < 0.05) and it was persistent as a plateau at least up to day 42 (Figure 1). Clearly, IL-10 overexpression was associated with sustained presence of C. burnetii in tissues and high levels of specific Abs, which was reminiscent of chronic Q fever.

### Decreased Granuloma Formation and Splenomegaly in macIL-10tg Mice

**Figure 2:**
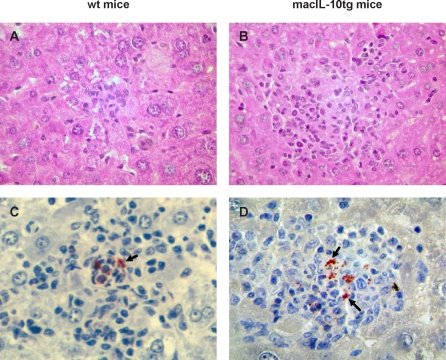
Liver Granulomas in WT and macIL-10tg Mice (A and B) Granulomas in the liver parenchyma of wt (A) and macIL-10tg (B) mice were revealed by hematoxylin-eosin staining. Representative micrographs of granulomas present at day 7 post-infection were shown (×400 original magnification). Note that granulomas were mainly composed of macrophages and were greater in macIL-10tg mice than in wt mice. (C and D) Liver sections from wt (C) and macIL-10tg (D) mice were deparaffinized and rehydrated, and C. burnetii organisms were revealed by immunohistostaining. Macrophages in inflammatory granulomas present in liver lobules were packed with granular immunopositive material (indicated using arrowheads). Immunopositive material was more abundant in macIL-10tg than in wt mice.

**Figure 3:**
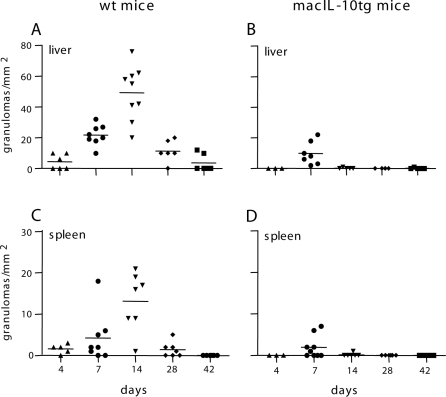
Granuloma Quantification Liver (A and B) and splenic (C and D) granulomas were numbered by image analysis. The results are expressed as the number of granulomas per square millimeter of tissues in wt (A and C) and transgenic (B and D) mice. Bars represent the median of values for each time point.

Granuloma formation is indicative of a protective immune response to C. burnetti and is defective in chronic Q fever [5]. While granulomas were easily identified in liver, they were merging with surrounding lymphoid tissue in spleen. In wt mice, granulomas detected in liver lobules and portobiliary spaces (Figure 2A) and the splenic red pulp (Figure S1) were mainly composed of Mφ with few lymphocytes and polymorphonuclear leukocytes. In transgenic mice, the composition of liver (Figure 2B) and splenic (Figure S1) granulomas was maintained, but their size was increased (compare Figure 2A and 2B). In addition, while liver (Figure 2C) and splenic (Figure S1) granulomas from wt mice were paucibacillary, liver (Figure 2D) and splenic (Figure S1) granulomas were multibacillary in macIL-10tg mice. The number of liver and splenic granulomas was quantified. In the liver (Figure 3A) and the spleen (Figure 3C) of wt mice, granulomas were detected at day 7 post-infection and their number increased at day 14 post-infection. Few granulomas were detected at day 28 post-infection. In macIL-10tg mice, granulomas were detected in liver (Figure 3B) and spleen (Figure 3D) at day 7 post-infection. However, they were no longer found at day 14 post-infection, in sharp contrast with wt mice. Histologic damages were not observed in the lungs of wt and macIL-10tg mice although bacterial DNA copies were found. Splenomegaly associated with C. burnetii infection was also different in wt and transgenic mice. In wt mice, the spleen weight was about 600 mg at day 7 post-infection versus 120 mg before infection. In macIL-10tg mice, the spleen weight moderately increased after 7 d (300 mg): the differences between wt and transgenic mice were significant (*p* < 0.05). These results showed that IL-10 inhibited granuloma formation and prevented splenomegaly in C. burnetii–infected mice. Again, the lack of granulomas in transgenic mice is reminiscent of chronic Q fever.

### Altered Activity of Mφ from macIL-10tg Mice

**Figure 4:**
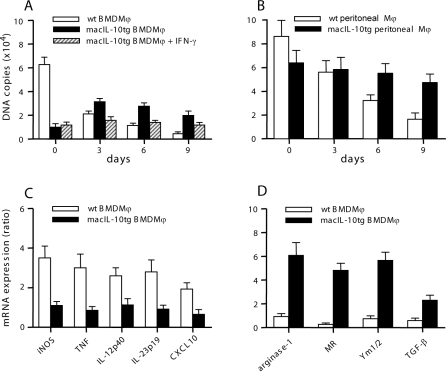
Mφ Responses to C. burnetii (A and B) BMDMφ (A) and peritoneal Mφ (B) were infected with C. burnetii for 4 h and cultivated for different periods. C. burnetii burden was measured by qPCR. The results are expressed as the mean number ± SD of C. burnetii DNA copies. (C and D) BMDMφ were stimulated by C. burnetii for 6 h, and transcripts for molecules corresponding to M1 phenotype (C) or M2 phenotype (D) were quantified by qRT-PCR. Results are expressed as the ratio of expression levels in C. burnetii-stimulated Mφ versus unstimulated Mφ (*n* = 5).

We next tested if the constitutive production of IL-10 by myeloid cells affects the microbicidal activity of Mφ toward C. burnetii and may account for bacterial persistence in transgenic mice. When bone marrow–derived Mφ (BMDMφ) were incubated with C. burnetii organisms at a bacterium-to-cell ratio of 100:1, the initial uptake of C. burnetii was significantly (*p* < 0.006) impaired in transgenic Mφ. After establishment of the infection, the number of bacterial DNA copies decreased in wt Mφ whereas it significantly (*p* < 0.005) increased in transgenic Mφ at day 3 post-infection. It remained significantly (*p* < 0.003) higher in transgenic Mφ than in wt Mφ at day 6 post-infection (Figure 4A). This difference may be related to impaired bacterial uptake by transgenic Mφ. C. burnetii uptake (about 2 × 10^4^ DNA copies) was rendered similar in wt and transgenic Mφ by using different infective doses of organisms (25:1 and 200:1 bacterium-to-cell ratios for wt and transgenic Mφ, respectively). While wt Mφ cleared C. burnetii organisms, transgenic Mφ allowed a moderate and transient replication of C. burnetii (unpublished data). The inability of transgenic Mφ to clear C. burnetii was not restricted to bone marrow–derived Mφ since wt peritoneal Mφ cleared C. burnetii whereas those of macIL-10tg mice remained infected until 9 d post-infection (Figure 4B). It is noteworthy that the defective uptake of C. burnetii by transgenic BMDMφ was corrected in peritoneal transgenic Mφ. Thus, IL-10 impaired microbicidal activity of Mφ in keeping with previous data using exogenous IL-10 stimulation of C. burnetii–infected Mφ [9]. We wondered if IFN-γ pre-treatment of transgenic BMDMφ restored their microbicidal competence. IFN-γ had no effect on bacterial uptake by Mφ from wt mice (unpublished data) or macIL-10tg mice (Figure 4A). IFN-γ did not change the microbicidal competence of wt Mφ (unpublished data). It specifically prevented C. burnetii replication in transgenic Mφ (*p* < 0.001 and *p* < 0.004 at days 3 and 6, respectively), but was unable to induce bacterial killing (Figure 4A). Finally, we studied the transcriptional profile of BMDMφ induced by C. burnetii. In the absence of infection, the overexpression of IL-10 in transgenic Mφ did not affect the expression of transcripts encoding molecules involved in the microbicidal activity of macrophages such as inducible NO synthase (iNOS) or molecules such as arginase-1 and mannose receptor (MR) (unpublished data). In wt Mφ, C. burnetii stimulated the expression of transcripts for iNOS, TNF, IL-12p40, IL-23p19, and CXCL-10 (Figure 4C) but did not stimulate arginase-1, MR, Ym1/2, and TGF-β (Figure 4D). This transcriptional pattern of Mφ is consistent with a microbicidal profile. By comparison, in transgenic Mφ, C. burnetii did not affect the expression of transcripts for iNOS, TNF, IL-12p40, IL-23p19, and CXCL-10 (Figure 4C) but stimulated the expression of transcripts for arginase-1, MR, Ym1/2, and TGFβ (Figure 4D). Hence, the constitutive overexpression of IL-10 in C. burnetii-stimulated Mφ is associated with a non-microbicidal transcriptional profile.

### Tissue Expression of Chemokines and Microbicidal Markers

**Figure 5:**
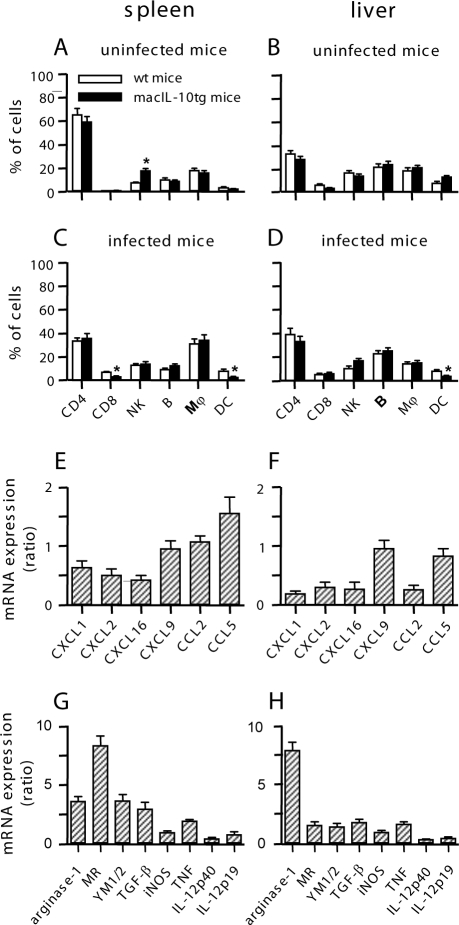
Tissue Recruitment of Leukocytes and Tissue Transcriptional Profile (A–D) The proportions of leukocyte subpopulations were determined by flow cytometry. Data are the mean ± SD of three mice/time point. *p < 0.05. (E–H) Transcripts encoding chemokines (E and H) and molecules corresponding to M1 and M2 phenotype (G and H) were quantified by qRT-PCR. Results are expressed as the ratio of expression levels in macIL-10tg mice and wt mice infected with C. burnetii for 14 d (*n* = 5).

The granuloma formation in spleen and liver, as a marker of efficient cell-mediated immunity, is associated with the recruitment of immunocompetent cells, which may be impaired in macIL-10tg mice. We investigated the distribution of leukocyte populations in spleen and liver by flow cytometry (Figure 5A–5D). In uninfected mice, only the percentage of NK cells was significantly (*p* < 0.05) increased in the spleen of macIL-10tg mice as compared to wt mice (16.8% versus 6.6%). At day 7 post-infection, less CD8^+^ T cells were recruited in the spleen from transgenic mice (2.9% versus 6.6% in wt mice; *p* < 0.002). The percentage of recruited DC was decreased in spleen (*p* < 0.01) and liver (*p* < 0.05) from macIL-10tg mice as compared to wt mice. We suggest that changes in tissue distribution of immunocompetent leukocytes are not sufficient to account for defective granuloma formation. As chemokines are required for leukocyte recruitment into granulomas and development of protective immunity [14], their expression in spleen and liver was assessed. In transgenic mice infected for 14 d, the splenic expression of transcripts for CXCL-1, CXCL-2, and CXCL-16 was decreased whereas that of CXCL-9, CCL-2, and CCL-5 was unaffected, as compared to infected wt mice (Figure 5E). In liver, the expression of mRNA for CXCL-1, CXCL-2, CXCL-16, and CCL-2 was markedly reduced, and that of CXCL-9 and CCL-5 was not affected (Figure 5F). The transcriptional pattern of spleen and liver chemokines found after 7 d of infection (unpublished data) was similar to that observed after 14 d. These results showed that the expression of chemokines was slightly altered in tissues. We finally wondered if the overexpression of IL-10 in mice resulted in a transition from a microbicidal to a non-microbicidal pattern in spleen and liver. The transcriptional pattern of molecules involved in the microbicidal machinery and M1/M2 polarization was similar in uninfected wt and transgenic mice (unpublished data). In 7-d-infected transgenic mice, the expression of arginase-1 mRNA was markedly increased in spleen (Figure 5G) and liver (Figure 5H), as compared to wt mice. The expression of MR was also markedly increased in spleen. Ym1/2 and TGF-β mRNA were also increased, but to a lesser extent. In contrast, in spleen and liver from transgenic mice, the expression of iNOS and TNF mRNA was not affected by infection and that of IL-12p40 and IL-23p19 was down-modulated. The transcriptional profile of tissues from mice infected for 14 d was similar to that found at day 7 post-infection (unpublished data). These results suggest that the overexpression of IL-10 is related to a tissue non-microbicidal pattern.

### Role of the Route of C. burnetii Infection in Bacterial Persistence

**Figure 6:**
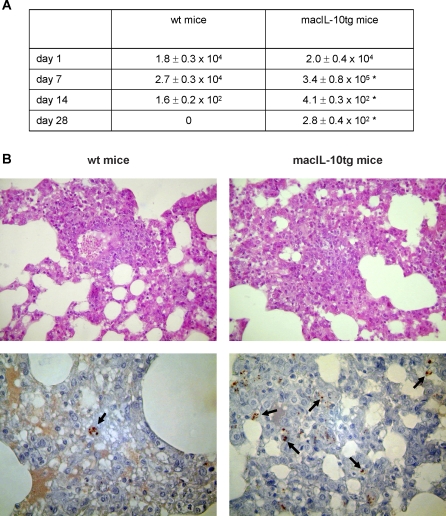
C. burnetii Infection and Pulmonary Lesions (A) Wild-type and macIL-10tg mice were injected with 5 × 10^5^ C. burnetii organisms by the intratracheal route. The number of bacterial DNA copies found in lungs was determined by qPCR. The results are expressed as mean ± SD of three mice per time point. *p < 0.05. (B) Pulmonary lesions in wt and macIL-10tg mice were revealed by hematoxylin-eosin staining. Top panels: representative micrographs of lesions present at day 7 post-infection were shown (×200 original magnification). Note a granulomatous interstial inflammation. Thickened alveolar walls were heavily infiltrated with mononuclear leukocytes, mainly macrophages. The size of granulomas was higher in transgenic mice than in wt mice. Bottom panels: pulmonary sections were deparaffinized and rehydrated, and C. burnetii organisms were revealed by immunohistostaining with hemalun counterstain. Macrophages in inflammatory granulomas present in interalveolar walls were packed with granular immunopositive material (indicated using arrowheads). Immunopositive material was more abundant in macIL-10tg than in wt mice. Magnification, ×400.

When wt and macIL-10tg mice were injected by the intratracheal route with 5 × 10^5^ organisms, mortality or morbidity was not observed up to 28 d. The lung infection was assessed by qPCR (Figure 6A). Sentinel mice were killed after 1 d of infection to determine the bacterial burden in lungs. There is no variation between wt and transgenic mice. In wt mice, the number of bacterial DNA copies slightly increased at day 7 post-infection, decreased at day 14, and no copies were detected at day 28 post-infection. In contrast, the number of bacterial DNA copies dramatically increased in transgenic mice at day 7 post-infection, demonstrating that C. burnetii organisms replicated within lungs. At day 14 post-infection, lung infection in transgenic mice decreased but remained significantly (*p* < 0.05) higher than in wt mice. At day 28 post-infection, a low number of bacterial DNA copies were still found in transgenic mice whereas wt mice had completely cured lung infection. We also found that the intratracheal route of C. burnetii inoculation was unable to induce liver and splenic infection in macIL-10tg mice (unpublished data).

We wondered whether C. burnetii replication within lungs was accompanied by histological changes. Inflammation was observed at days 7 and 14 post-infection in wt and transgenic mice. Inflammatory infiltrates were largely confined within the walls of the alveoli. Infiltrates were often organized as granulomatous interstitial inflammation and consisted mainly of macrophages with few lymphocytes (Figure 6B). Granulomas of variable diameter scattered throughout the interalveolar walls of the lung parenchyma. However, the size of lung granulomas was lower in wt mice than in transgenic mice. The bronchoalveolar air spaces were relatively free of cellular exsudates, but some are filled with rare alveolar macrophages. Neither necrosis of the lining alveolar epithelium nor suppuration was observed. These findings are consistent with a mixed interstitial and mild alveolar mononuclear cell pneumonia. Finally, we studied the pulmonary localization of C. burnetii at day 7 post-infection. Organisms were seen as granular immunopositive material in few alveolar macrophages (unpublished data). They were mainly found in the granulomatous interstitial inflammation within cells that had the morphology of macrophages. In wt mice, interalveolar wall granulomas were paucibacillary. In contrast, they were multibacillary in transgenic mice (Figure 6B).

## Discussion

In humans, C. burnetii infection can be asymptomatic or result in acute or chronic disease [5,6]. Mice are usually used as animal model for acute Q fever [15]. Different animal models of chronic Q fever are based on dramatic immunosuppression [16,17], but the chronic evolution of Q fever is not associated with severe immunosuppression but it requires IL-10 in humans [7,8]. In fact, an appropriate mouse model for the study of chronic Q fever is lacking. We wondered if the constitutive overexpression of IL-10 in the myeloid compartment from genetically modified mice would reproduce aspects of the human disease. When C. burnetii was injected by the intraperitoneal route, organisms were rapidly cleared in wt mice while macIL-10tg mice maintained bacterial loads for at least 42 d. The sustained presence of C. burnetii in tissues was associated with high circulating levels of specific IgG as in Q fever endocarditis [5]. The anti-C. burnetii IgG2a were prominent in macIL-10tg mice (unpublished data), which is consistent with increased levels of IgG1 and IgG3 in human Q fever [18]. C. burnetii persistence is also consistent with the role of IL-10 in latent tuberculosis [19].

As humans are most commonly infected with C. burnetii through inhalation of parturient secretions from infected animals [6], wt and transgenic mice were infected by the intratracheal route. The constitutive overexpression of IL-10 dramatically increased lung infection. The pulmonary lesions consisting of mixed interstitial and mild alveolar mononuclear cell pneumonia were previously published in a model of aerosol infection [20]; they were more pronounced in macIL-10tg mice than in wt mice. These results combined with a previous publication [20] emphasized the role of the route of inoculation in C. burnetii infection in mice as well as in humans.

The second important feature of chronic Q fever is the lack of granulomas that are replaced by mononuclear cell infiltrates [6]. In macIL-10tg mice, C. burnetii infection was associated with decreased formation of liver and splenic granulomas without alteration of their organization. This finding was specific since granuloma formation was normal in BCG-infected macIL-10tg mice [13]. The mechanism of defective microbicidal response to C. burnetii manifested as bacterial persistence and defective granuloma formation may involve innate and/or adaptive immunity [21]. Specifically, the immune response in granulomas may be affected by defective recruitment of immune effectors and/or polarization of protective immune response toward non-microbicidal immune response. First, we recently reported that transendothelial migration of mononuclear cells is impaired in chronic Q fever; this deficiency is corrected when IL-10 is neutralized [22]. However, we found that the transmigration of murine mononuclear cells was similar in wt and macIL-10tg mice (unpublished data). Second, we hypothesized that IL-10 overexpression may impair trafficking of immune cells, thus preventing cell recruitment in granulomas: it was not the case. The percentage of T cells, B cells, Mφ, and DC was similar in spleen and liver from uninfected wt and macIL-10tg mice, as described elsewhere [13]; only the percentage of NK cells was increased in the spleen of transgenic mice, which is consistent with the ability of IL-10 to stimulate NK cells [23]. C. burnetii infection decreased the proportion of DC in transgenic mice. C. burnetii is known to impair in vitro activation and maturation of DC [24], but it seems unlikely that DC impairment is sufficient to prevent splenomegaly and granuloma formation in spleen and liver. These findings suggest that the recruitment of immune effectors was not significantly impaired in mice constitutively overexpressing IL-10. Rather, they suggest the recruitment of non-microbicidal immune cells into tissues, which may account for defective killing of C. burnetii and granuloma formation. This is supported by our in vitro findings with Mφ. Mφ from macIL-10tg mice allowed C. burnetii replication whereas C. burnetii was killed by wt Mφ. Impaired microbicidal competence was not corrected by IFN-γ. This finding may be critical to understand bacteria persistence in mice and humans despite an apparent efficient immune response. As microbicidal activity of Mφ is associated with transcriptional pattern of classical activation by cytokines and microbial products [25], we studied the transcriptional pattern of C. burnetii-stimulated Mφ. While wt Mφ exhibited a classical inflammatory transcriptional pattern, Mφ from macIL-10tg mice exhibited a non-microbicidal pattern in which iNOS and inflammatory cytokines were not induced and arginase-1 and TGF-β were stimulated. The differential expression of arginase-1 and iNOS mRNA may correlate with the ability of Mφ to control C. burnetii replication since previous reports have shown increased susceptibility of iNOS-deficient mice toward C. burnetii [26]. Similarly, the gradual decrease in iNOS expression is correlated with the transition from latent tuberculosis to progressive pulmonary tuberculosis [27]. Hence, the balance between iNOS and arginase-1 may be essential to regulate inflammation and microbicidal response to infectious agression [28]. The responses of host tissues to C. burnetii infection may also reflect Mφ polarization toward type 2 immune response. Indeed, the expression of CXCL-1, CXCL-2, CXCL16, and CCL-2 was reduced in liver from macIL-10tg mice, and it is established that CXCL-1, CXCL-2, and CCL-2 are regulated by IL-10 [29]. In C. burnetii–infected transgenic mice, the expression of arginase-1, MR, Ym1/2, and TGF-β was increased in spleen, and to a lesser extent, in liver, whereas the expression of iNOS, IL-12, and IL-23 was down-modulated. The combination of high expression levels of arginase-1 and Ym-1 and low levels of iNOS in tissues is consistent with M-2 type phenotype described in Mφ [30].

In conclusion, IL-10 was essential for sustained C. burnetii burden in tissues, high levels of Abs, and impaired granuloma formation, three characteristics of chronic Q fever. IL-10 affected tissue inflammatory gene expression and Mφ polarization and may contribute to defective granuloma formation. Hence, constitutive overexpression of IL-10 is an experimental model of persistent infection by C. burnetii that could serve as a platform for anti-C. burnetii drug or immunotherapy development.

## Materials and Methods

### Infection of mice.

C. burnetii (Nine Mile strain) organisms in phase I (virulent organisms) and phase II (avirulent organisms) were obtained as previously described [31]. Control mice (FVB background) and macIL-10tg mice were kept in a specific pathogen-free mouse facility and handled according to the rules of Décret N° 87–848 du 19/10/1987, Paris. The experimental protocol have been reviewed and approved by the Institutional Animal Care and Use Committee of the Université de la Méditerranée. Female mice were infected with 5 × 10^5^ C. burnetii organisms by intraperitoneal route, and, in some experiments, by intratracheal route. The clinical status of mice was recorded daily. Mice were killed after different infection times until day 60. Organs were aseptically excised, and tissue samples were embedded in paraffin or frozen at −80 °C.

### C. burnetii detection*.*

Tissues (10–25 mg) were incubated with 180 μL of lysis buffer and 20 μL of proteinase K, and DNA was extracted by using the QIAamp DNA MiniKit (Qiagen). Quantitative real-time PCR (qPCR) was performed with the LightCycler system (Roche) and carried out with 5-μl DNA samples and specific primers, as previously described [32]. In each qPCR run, a standard curve was generated using serial dilution ranging from 10^8^ to 10^4^ copies of the intergenic spacer region. Tissue sections were deparaffinized in xylene and rehydrated in graded alcohol. Bacteria were revealed using the Immunostain-Plus kit (Zymed), as previously described [33]. In brief, tissue sections were incubated with rabbit anti-C. burnetii Abs, or normal rabbit serum as control, used at a 1:2,000 dilution and secondary Abs conjugated with peroxidase.

### Antibody determination.

Slides with smears of formaldehyde-inactivated organisms in phase I or phase II were incubated with serial dilutions of serum from infected mice, as described elsewhere [32]. Bacteria were labeled with FITC-conjugated goat Abs directed against mouse IgG (Beckman Coulter) and rat Abs against mouse IgG1, IgG2a, IgG2b, and IgG3 (BD Biosciences) at a 1:100 dilution for 30 min. After washing, the slides were examined by fluorescence microscopy. The starting dilution for the serum sample was 1:25, and samples were titered to end point.

### Determination of granulomas.

The 5-μm sections of paraffin-embedded tissues were stained with hematoxylin-eosin to assess the presence of granulomas, defined as collections of ten or more macrophages and lymphocytes. Their number was determined after whole optical examination of at least three tissue sections. They were quantified by image analysis [32]. The results are expressed as the number of granulomas found per surface unit (mm^2^).

### Mφ studies.

Bone marrow precursor cells were cultured with 15% L-cell-conditioned medium. After a 7-d culture, more than 90% of cells were Mφ, as determined by morphological and phagocytic criteria. BMDMφ were scrapped and plated in 24-well tissue culture dishes at a density of 2 × 10^5^ per well. In some experiments, Mφ were treated with IFN-γ (100 UI/mL, R&D Systems) for 1 d before infection. To determine bacterial phagocytosis, Mφ were incubated with C. burnetii for 4 h, washed to eliminate free organisms (day 0), and the number of bacterial DNA copies was assessed by qPCR, as described above. Mφ were then cultivated for 9 d, and bacterial replication was assessed. In some experiments, peritoneal macrophages were used instead BMDMφ. Measurements of cytokines, iNOS, arginase-1, MR, and Ym1/2 were determined by quantitative real-time RT-PCR (qRT-PCR) as follows. C. burnetii organisms (100:1 bacterium-to-cell ratio) were added to Mφ for 6 h and total RNA was extracted. The cDNA synthesis was carried out with primers using the primer3 tool available at the following website: http://frodo.wi.mit.edu/ (Table 1). Reverse transcriptase was omitted in negative controls. The fold change in target gene cDNA was determined relative to the β-actin endogenous control.

**Table 1:**
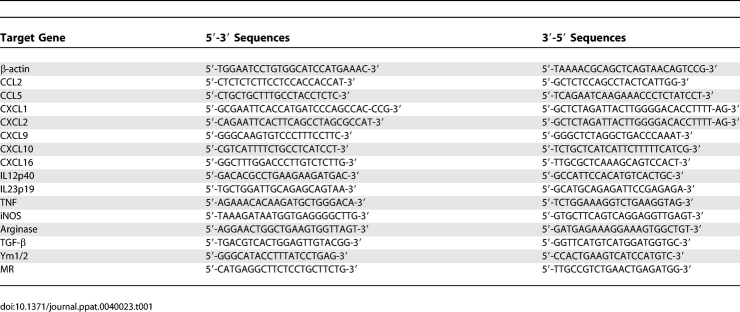
Nucleotide Sequences of Oligonucleotide Primers

### Flow cytometric analysis of tissue leukocytes.

Splenic and liver cells were deposited on Ficoll gradient (MSL, Eurobio), as recently described [32]. Purified leukocytes were stained with fluorophore-conjugated monoclonal Abs (mAbs, 1/100 dilution), fixed in 2% paraformaldehyde, and analyzed using a FACSCalibur instrument (BD Biosciences). At least 10^4^ cells were analyzed per sample. The following mAbs (BD Biosciences) were used for immunophenotyping leukocytes: PE-conjugated rat anti-mouse CD4^+^ or CD8^+^ T cells, FITC-conjugated rat anti-mouse NK1.1 (NK cells), allophycocyanin-conjugated rat anti-mouse B220 (B cells), allophycocyanin-conjugated rat anti-mouse CD11b (Mac-1 chain; Mφ), and FITC-conjugated rat anti-mouse CD11c (dendritic cells, DC). In all cases, isotype-matched fluorophore-conjugated IgGs were used.

### Tissue transcriptional profile.

The presence of chemokines and molecules corresponding to M1 phenotype or M2 phenotype were quantified by qRT-PCR, as described elsewhere [32]. Briefly, RNA was extracted, and the cDNA synthesis was carried out with 10 ng of total RNA, oligo (dT) primer, and M-MLV reverse transcriptase (Invitrogen). PCR was performed using the LightCycler. The primer sequences were designed using the website designed above. The fold change in target gene cDNA was determined as the ratio of expression levels in macIL-10tg mice and wt mice infected with C. burnetii.

### Statistical analysis.

Results, given as median or mean ± SD, were compared with the Mann-Whitney *U* test. Differences were considered significant when *p* < 0.05.

## Supporting Information

Figure S1 Splenic Granulomas in WT and macIL-10tg Mice Granulomas in the red pulp of spleens and C. burnetii material were revealed by hematoxylin-eosin staining and immunohistostaining, respectively. Representative micrographs of granulomas present at day 7 post-infection were shown (×400 original magnification). Note that granulomas were mainly composed of macrophages and were greater in macIL-10tg mice than in wt mice. Granular immunopositive material is indicated using arrowheads. Immunopositive material was more abundant in macIL-10tg than in wt mice.
(4.8 MB DOC)
